# Hypoxia Differentially Regulates Ferroptosis Sensitivity and Tumor Cell-Intrinsic Type I Interferon Signaling in Pancreatic Ductal Adenocarcinoma Cells

**DOI:** 10.3390/ijms27146397

**Published:** 2026-07-18

**Authors:** Shubhankar Das, Ayda Shah Mahmood, Salem Chouaib

**Affiliations:** 1 Thumbay Research Institute for Precision Medicine, Gulf Medical University, University Street, Street #1, Ajman P.O. Box 4184, United Arab Emirates; dr.shubhankar@gmu.ac.ae (S.D.); ayda@gmu.ac.ae (A.S.M.); 2 Integrative Tumor Immunology and Immunotherapy, Gustave Roussy, University Paris-Saclay, INSERM UMR 1186, F-94805 Villejuif, France

**Keywords:** pancreatic ductal adenocarcinoma, ferroptosis, hypoxia, innate immune signaling, tumor microenvironment

## Abstract

Ferroptosis has emerged as a promising strategy to overcome resistance to conventional cancer therapies. Pancreatic ductal adenocarcinoma (PDAC) is characterized by hypoxia, therapy resistance, and an immunosuppressive microenvironment. Although hypoxia is likely to influence ferroptosis susceptibility and the associated inflammatory pathways that regulate antitumor immunity, their impact on ferroptosis sensitivity and innate immune responses remains poorly understood. In this study, we investigated the effects of hypoxia on the induction of ferroptosis and immune-related signaling in PDAC cell lines. We examined how hypoxia affects the responses of Panc-1, BxPC3, and Capan-1 cells to the ferroptosis inducers RAS-selective lethal 3 (RSL3)/Imidazole ketone erastin (IKE) under normoxic and hypoxic (0.1% O_2_) conditions. Cell viability assays were used to assess ferroptosis sensitivity, and rescue experiments were performed using liproxstatin-1 (LIP). Gene expression analysis was conducted to evaluate changes in immune, interferon, inflammatory, and hypoxia-related genes following ferroptosis induction. Panc-1 cells were the most sensitive, whereas Capan-1 cells were resistant, particularly under hypoxia. Ferroptosis triggered cell line-specific responses involving interferon signaling, inflammation, and stress pathways. Panc-1 cells showed over-expression of *RIG-I, MAVS, IRF3/7/9, STAT1/2,* and *CXCL10*, particularly under hypoxia, indicating activation of Type I interferon (IFN)-associated transcriptional program. BxPC3 cells demonstrated broader cytokine induction, including *IL-8*, *CCL2*, *CXCL2*, *GM-CSF*, and *IL-11*, whereas Capan-1 cells were minimally responsive. Hypoxia also increased *ANGPTL4* and *GDF15* expression following ferroptosis induction. These findings show that hypoxia differentially affects ferroptosis sensitivity and immune responses in PDAC, revealing complex interactions among ferroptosis, innate immunity, and the tumor microenvironment.

## 1. Introduction

Pancreatic ductal adenocarcinoma (PDAC) remains one of the most lethal malignancies worldwide and is characterized by aggressive progression, profound resistance to chemotherapy, and limited responsiveness to immunotherapy [1,2]. Unlike immunogenic tumors, PDAC exhibits sparse cytotoxic lymphocyte infiltration, dense stromal desmoplasia, and a highly immunosuppressive tumor microenvironment (TME) that collectively impair effective anti-tumor immune surveillance [3,4]. Current therapeutic strategies targeting immune checkpoints have shown only modest clinical benefit in PDAC, underscoring the need to identify tumor-intrinsic stress pathways to enhance tumor immunogenicity and improve immune-mediated tumor clearance [5,6].

Ferroptosis has emerged as a promising therapeutic vulnerability in cancer. Ferroptosis is a regulated, iron-dependent form of cell death driven by excessive lipid peroxidation following disruption of glutathione-dependent antioxidant defenses [7,8]. Pharmacologic inhibition of glutathione peroxidase 4 (GPX4) with RAS-selective lethal 3 (RSL3) or blockade of the cystine/glutamate antiporter system Xc^−^ using erastin derivatives such as imidazole ketone erastin (IKE) induces ferroptotic death in multiple tumor types, including PDAC [9]. Beyond its cytotoxic effects, ferroptosis is increasingly recognized as an immunologically active form of stress that influences inflammatory signaling, cytokine secretion, and tumor–immune interactions [10,11]. Oxidative lipid damage, mitochondrial dysfunction, and damage-associated molecular patterns generated during ferroptosis may activate innate immune pathways and modulate recruitment or activation of immune cells within the TME [12,13].

Type I interferon (IFN) signaling represents a central pathway linking cellular stress to innate immune activation. Cytosolic RNA-sensing pathways involving RIG-I/MAVS–Type I IFN–JAK/STAT axis regulate transcription of interferon-stimulated genes (ISGs), including *ISG15*, *OAS1*, *MX1*, and *CXCL10*, which collectively promote antiviral responses, antigen presentation, and recruitment of cytotoxic immune cells [14,15]. In cancer, activation of tumor-intrinsic interferon signaling has been associated with enhanced immune recognition and improved responsiveness to immunotherapy, whereas defects in this pathway contribute to immune evasion [16]. Recent evidence suggests that ferroptosis-associated oxidative stress may intersect with innate immune signaling pathways; however, the extent to which ferroptosis modulates interferon-associated responses in PDAC remains poorly understood.

A defining feature of PDAC biology is severe hypoxia and extensive stromal fibrosis. Hypoxia can arise from reduced oxygen delivery due to impaired blood flow, vascular abnormalities, or limited oxygen diffusion [17]. In PDAC, hypoxia is frequently observed because of rapid tumor growth that, in turn, exceeds the capacity of the existing vasculature, while newly formed tumor vessels are often structurally abnormal and inefficient in oxygen delivery. This leads to a TME with extremely low oxygen tension (<2.5 mm Hg) [18]. Hypoxic stress activates hypoxia-inducible factors (HIFs), which regulate adaptive responses in metabolism, angiogenesis, and redox homeostasis, thereby conferring a survival advantage on cells [19].

Hypoxia profoundly alters tumor metabolism, oxidative stress responses, immune-cell infiltration, and inflammatory signaling within the TME. Importantly, hypoxia can exert complex and context-dependent effects on ferroptosis. While oxygen availability contributes to lipid peroxidation, hypoxia-induced metabolic adaptation, antioxidant activation, and lipid remodeling may either enhance or suppress susceptibility to ferroptosis, depending on the cellular context [20]. Hypoxia is also known to regulate immune signaling pathways, including suppression of interferon-associated transcriptional responses and promotion of immunosuppressive cytokine networks [21,22]. Consequently, the interaction between ferroptosis and hypoxia may critically determine whether ferroptotic stress promotes anti-tumor immunity or instead contributes to tumor adaptation and immune evasion.

In the present study, we investigated the impact of ferroptosis induction under normoxic and hypoxic conditions in multiple PDAC cell lines exhibiting differential ferroptosis sensitivity. As PDAC is a genetically and phenotypically heterogeneous disease, we used multiple PDAC cell lines with distinct molecular characteristics to evaluate ferroptosis-associated responses and innate immune signaling under normoxic and hypoxic conditions. PANC-1, BxPC-3, and Capan-1 cells represent commonly used PDAC models with distinct genetic and phenotypic features [23,24,25]. PANC-1 cells harbor mutant KRAS (G12D) and mutant TP53, exhibit a mesenchymal-like phenotype, and are characterized by relatively high invasive capacity and metabolic plasticity. BxPC-3 cells are KRAS wild-type, harbor mutant TP53, and display a more epithelial phenotype, representing a distinct molecular subtype of PDAC. Capan-1 cells harbor mutant KRAS (G12V) and mutant TP53, exhibit epithelial characteristics, and were originally derived from a metastatic PDAC background. These genetic differences influence basal metabolic states, antioxidant capacity, stress adaptation mechanisms, and sensitivity to therapeutic stress. Importantly, variation among these models provides an opportunity to examine whether hypoxia- and ferroptosis-induced modulation of type I interferon signaling represents a common PDAC response or is influenced by the intrinsic molecular context of individual tumor cells.

Using RSL3 and IKE, we evaluated ferroptotic susceptibility and characterized the expression of innate immune, interferon-response, inflammatory, and hypoxia-associated genes following ferroptosis induction. We demonstrate that ferroptosis induces highly distinct cell line-specific transcriptional responses in PDAC cells. Panc-1 cells exhibited strong activation of interferon and antiviral signaling pathways, including *RIG-I*, *MAVS*, *IRF1/3/7/9*, *STAT1/2*, and *CXCL10*, particularly under hypoxic conditions. In contrast, BxPC3 cells exhibited a broader induction of inflammatory cytokines and chemokines, including *IL-8*, *CCL2*, *CXCL2*, *GM-CSF*, and *IL-11*, whereas Capan-1 cells remained largely resistant to both ferroptosis and transcriptional reprogramming. Additionally, ferroptosis combined with hypoxia enhanced the expression of stress-associated immunomodulatory genes, including *ANGPTL4* and *GDF15.*

Collectively, these findings demonstrate that hypoxia differentially regulates ferroptosis sensitivity and ferroptosis-associated immune signaling programs in PDAC. Our results further suggest that ferroptosis may exert both immunostimulatory and immunosuppressive effects, depending on the cellular and hypoxic context, thereby influencing the inflammatory composition and immune landscape of PDAC. These observations provide mechanistic insight into the complex interplay among ferroptosis, hypoxia, and innate immune signaling and support the development of combinatorial therapeutic strategies that integrate ferroptosis induction with immune- and hypoxia-targeting approaches in PDAC.

## 2. Results

### 2.1. PDAC Cell Lines Exhibit a Differential Response to Ferroptosis in Normoxia and Hypoxia

**Figure 1:**
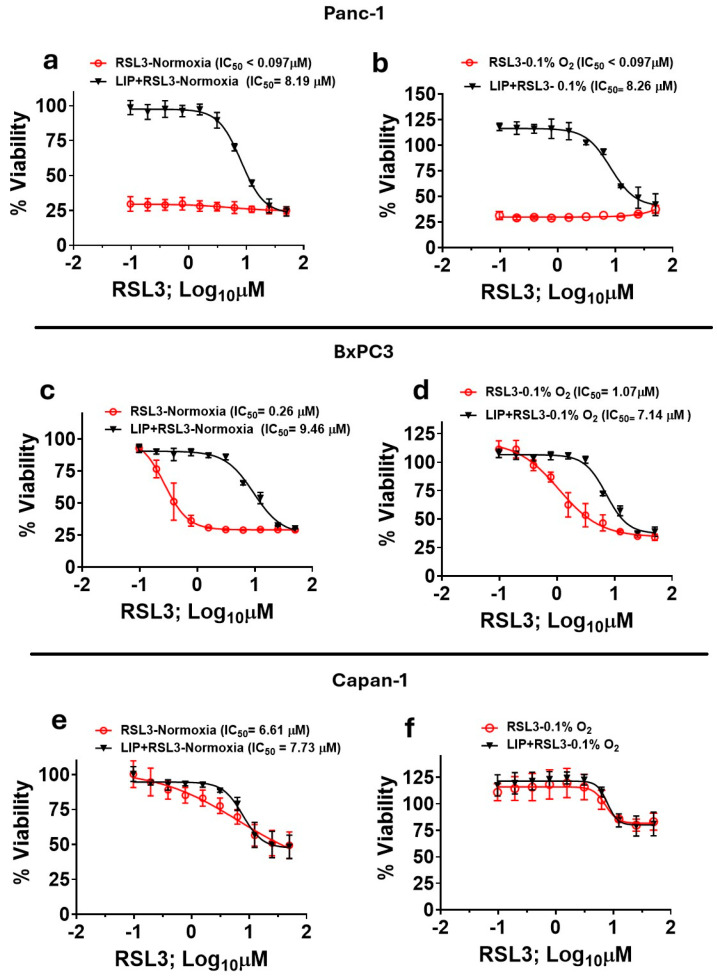
Effect of RSL3 on PDAC cell viability under normoxic and hypoxic (0.1% O_2_) conditions. Dose–response viability assays were performed in PDAC cell lines treated with increasing concentrations of the ferroptosis inducer RSL3 under normoxia or hypoxia (0.1% O_2_) for 72 h. Panels (**a**,**b**) represent Panc-1 cells cultured under normoxic and hypoxic conditions, respectively; panels (**c**,**d**) represent BxPC3 cells under normoxic and hypoxic conditions, respectively; and panels (**e**,**f**) represent Capan-1 cells under normoxic and hypoxic conditions, respectively. Cell viability was normalized to untreated controls and plotted as percentage viability across RSL3 concentrations.

In this set of experiments, we determined the differential sensitivity of the PDAC cell lines Panc-1, BxPC3, and Capan-1 to the ferroptosis inducers IKE and RSL3. To confirm that the observed cell death was ferroptosis-dependent, Liproxstatin-1 (LIP), a selective ferroptosis inhibitor, was used. LIP suppresses ferroptosis by acting as a lipid radical-trapping antioxidant that prevents iron-dependent lipid peroxidation and subsequent membrane damage. Therefore, LIP-mediated reversal of ferroptotic cell death indicates that the observed phenotype is specifically associated with lipid peroxidation-driven ferroptotic mechanisms. Treatment of PDAC cell lines with the ferroptosis inducer RSL3 led to distinct viability responses under normoxic and hypoxic conditions. Panc-1 cells, both in normoxia and hypoxia, exhibited markedly increased sensitivity to RSL3, such that the lowest concentration of 90 nM resulted in approximately 30% cell viability both in normoxia and hypoxia, indicating enhanced ferroptotic susceptibility independent of oxygen conditions. This was rescued by conditioning the cells with 500 nM of LIP (Figure 1a,b). In BxPC3 cells, treatment with RSL3 resulted in loss of viability, with IC_50_ values of 0.26 µM and 1.07 µM in normoxia and hypoxia, respectively. As with Panc-1, conditioning BxPC3 cells with LIP reduced viability in both normoxia and hypoxia, with IC_50_ values increasing to 9.46 µM and 7.14 µM in normoxia and hypoxia, respectively (Figure 1c,d). In contrast, Capan-1 cells demonstrated greater resistance to RSL3 than Panc-1 and BxPC3 cells, in both normoxia and hypoxia. The IC_50_ for RSL3 in Capan-1 cells was 6.61 µM under normoxic conditions. Cells conditioned with LIP and then treated with RSL3 showed increased cell viability under normoxia, with an IC_50_ of 7.73 µM. Under hypoxic conditions, Capan-1 cells exhibited marked resistance to RSL3 when compared to the other cell lines used in this study. In fact, under hypoxia, cell viability was greater than 75% even at the highest tested concentration of 25 µM of RSL3. Overall, these findings suggest that hypoxia potentiates RSL3-induced cytotoxicity in a cell line-dependent manner, with Panc-1 and BxPC3 cells exhibiting greater sensitivity to hypoxia-associated ferroptosis than Capan-1 cells.

**Figure 2:**
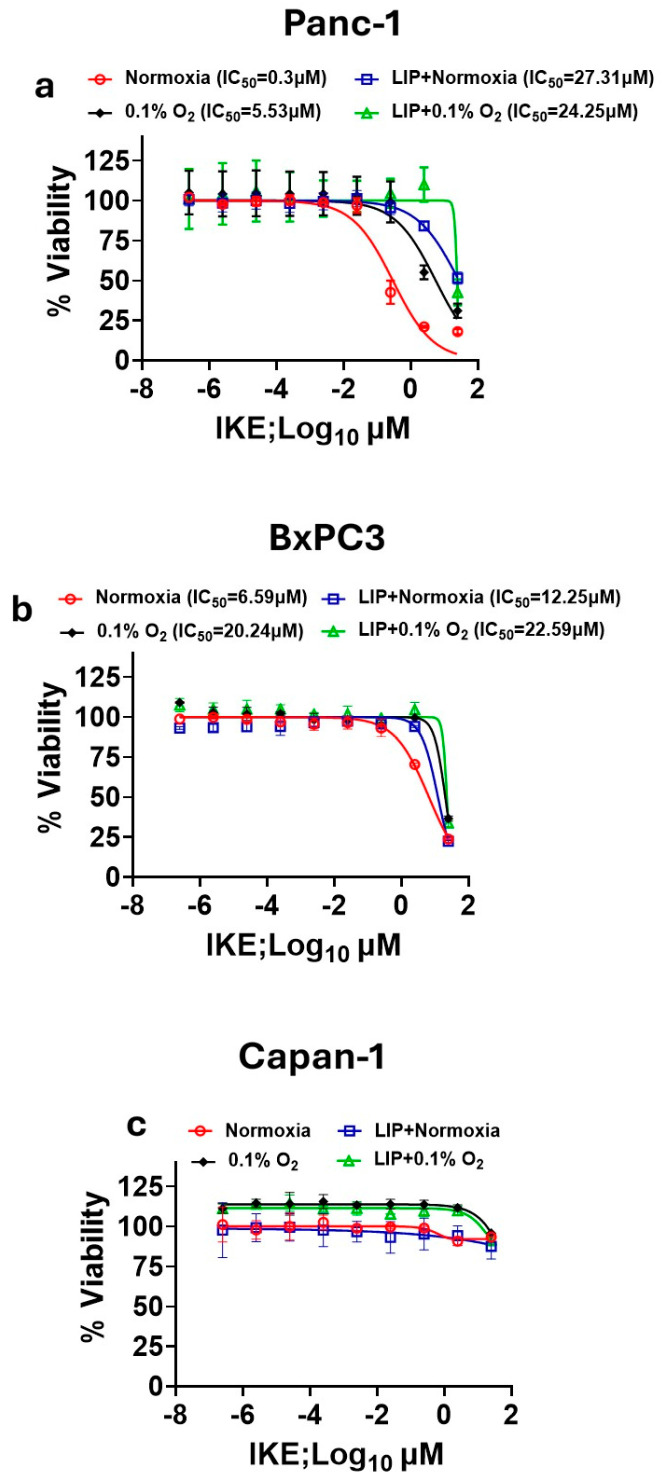
Effect of IKE on PDAC cell viability under normoxic and hypoxic (0.1% O_2_) conditions. Dose–response viability assays were performed in PDAC cell lines treated with increasing concentrations of the ferroptosis inducer IKE under normoxia or hypoxia (0.1% O_2_) for 72 h. (**a**) Graph shows Panc-1 cells cultured under normoxic and hypoxic conditions, respectively; (**b**) graph indicates BxPC3 cells under normoxia and hypoxia, respectively; and (**c**) graph represents Capan-1 cells under normoxic and hypoxic conditions, respectively. Cell viability was normalized to untreated controls and plotted as percentage viability across IKE concentrations.

Correspondingly, all three PDAC cell lines used in this study showed similar differential sensitivity to IKE, too, Panc-1 cells being most sensitive and Capan-1 being most resistant. As indicated in Figure 2a, Panc-1 cells showed IC_50_ values of 0.3 and 5.53 µM in normoxia and hypoxia, respectively, upon treatment with different concentrations of IKE alone. This was rescued by conditioning the cells with 500 nM LIP (Figure 2a), yielding IC_50_ values of 27.31 and 24.25 µM. In BxPC3 cells, treatment with IKE altered cell viability, with IC_50_ values of 6.65 µM and 20.24 µM in normoxia and hypoxia, respectively. As with Panc-1, conditioning BxPC3 cells with LIP reduced viability in both normoxia and hypoxia, with IC_50_ values increasing to 12.25 µM and 22.29 µM, respectively (Figure 2b). In contrast, Capan-1 cells demonstrated greater resistance to IKE than Panc-1 and BxPC3 cells, in both normoxia and hypoxia. In fact, under normoxia or hypoxia, there was no cell death (Figure 2c). Overall, these findings suggest that, compared to RSL3, IKE is less toxic to cells in both normoxia and hypoxia, and that Capan-1 is highly resistant to IKE-mediated ferroptotic death.

### 2.2. Hypoxia Differentially Modulates Ferroptosis-associated Transcriptional Responses of Innate Immunity and Inflammation in PDAC Cells

Since ferroptosis can influence tumor–immune interactions, particularly NK-cell activity, we analyzed the expression of cytokines, chemokines, interferon-response genes, and stress-associated mediators implicated in innate immune activation and immune-cell recruitment following RSL3 and IKE treatment under normoxia and hypoxia. The genes investigated comprised the following: (A) Type I IFN signaling *(RIG-I*, *MAVS*, *IRF1*, *IRF3*, *IRF7*, *IRF9*, *STAT1*, *STAT2*, *MX1*, *OAS1*, *ADAR-p150*, *CXCL10*), (B) hypoxia/stress-associated mediators linked to tumor adaptation and immune modulation (*TGFβ*, *ANGPTL4*, *GDF15*), and (C) dual-role mediators that are context-dependent (*IL-8*, *CCL2*, *CXCL2*, *GM-CSF*, *IL-11*). The gene panel captures key components of type I interferon signaling, hypoxia stress adaptation, and inflammatory networks, based on their roles in innate immunity, tumor progression, and cellular stress responses [21,26,27,28,29]. We chose the 36 h time point to measure the changes in the expression of the above-mentioned genes, given the varying sensitivities of Capan-1, BxPC-3, and PANC-1 cells to IKE- and RSL3-induced ferroptosis. This timing captures sustained responses in ferroptosis-related genes while avoiding excessive cell death that could compromise RNA quality and gene expression analysis.

#### 2.2.1. Type I IFN Signaling

**Figure 3:**
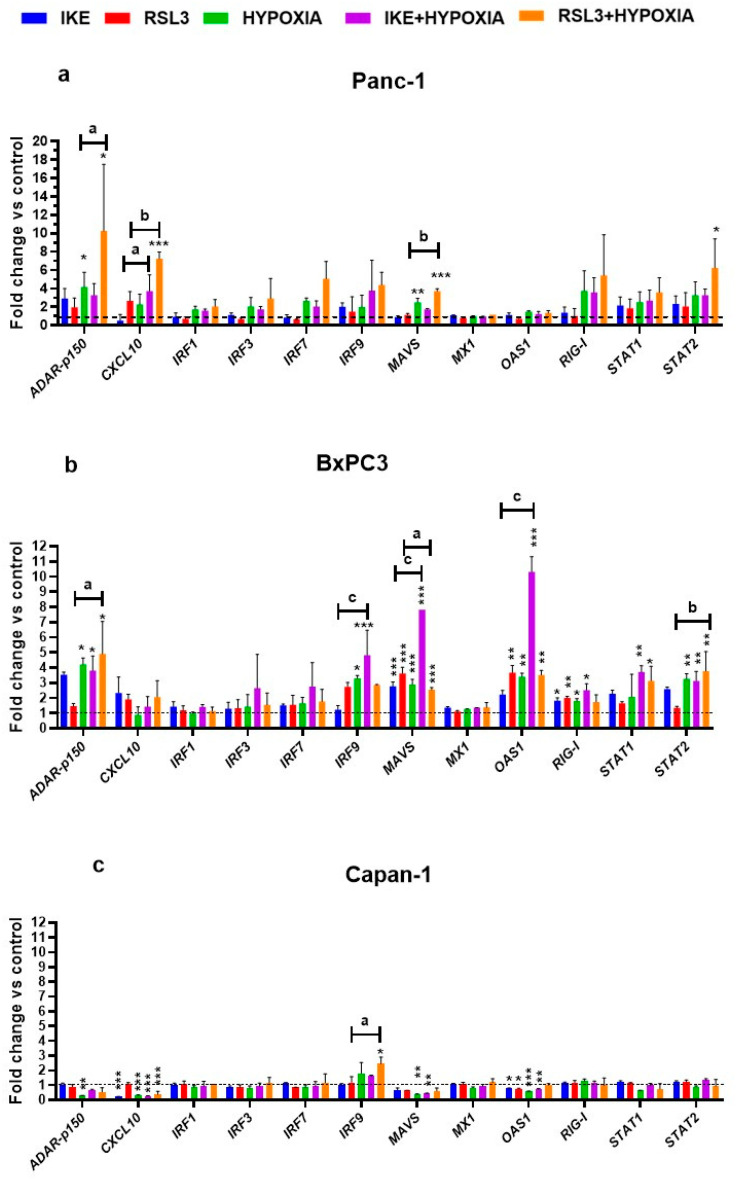
Effect of ferroptosis induction on innate immune and interferon-response gene expression in PDAC cell lines (**a**) Panc-1, (**b**) BxPC3, and (**c**) Capan-1 cells. Relative mRNA expression of the indicated genes was evaluated following treatment with RSL3 (100 nM) or IKE (1 µM) in normoxia or 0.1% hypoxia, measured at 36 h. Statistical significance: * *p* < 0.05, ** *p* < 0.01, *** *p* < 0.001 when compared to untreated control. ^a^ *p* < 0.05, ^b^ *p* < 0.01, ^c^ *p* < 0.001 when compared to IKE/RSL3 treated in normoxia. No symbol indicates no statistical significance.

The impact of ferroptosis on innate immune signaling in Panc-1, BxPC3, and Capan-1 cells treated with RSL3 or IKE under normoxic and hypoxic conditions has distinct cell line-specific responses. Among the three PDAC cell lines, Panc-1 cells demonstrated the strongest transcriptional activation of innate immune signaling pathways following ferroptosis induction. Treatment with RSL3 or IKE under normoxia increased the expression of *ADAR-p150*, *CXCL10*, *IRF1*, *IRF3*, *IRF7*, *IRF9*, *MAVS*, *RIG-I*, *STAT1*, and *STAT2*, with substantially greater induction under hypoxic conditions. These findings indicate that hypoxia potentiates ferroptosis-associated interferon and antiviral signaling in Panc-1 cells (Figure 3a).

As shown in Figure 3b, BxPC3 cells showed a similar but comparatively weaker response pattern. Ferroptosis induction in combination with hypoxia enhanced the expression of several innate immune genes; however, the magnitude of induction was lower than that observed in Panc-1 cells. Notably, *IRF3*, *IRF7*, *IRF9*, *OAS1*, and *MAVS* were more strongly expressed in BxPC3 cells under hypoxia combined with ferroptosis induction, suggesting preferential activation of viral RNA sensing and interferon-amplification pathways in this cell line. In contrast, *ADAR-p150* and *CXCL10* expression levels were markedly higher in Panc-1 cells than in BxPC3 cells, indicating a stronger inflammatory and interferon-associated phenotype in Panc-1 cells. Capan-1 cells displayed minimal transcriptional responsiveness to ferroptosis induction. Most genes analyzed remained at levels comparable to those in untreated controls across the normoxic and hypoxic treatment groups. However, a notable exception was *IRF9*, which was significantly overexpressed specifically in the RSL3 + hypoxia group, suggesting selective activation of interferon-stimulated transcriptional machinery under combined oxidative and hypoxic stress (Figure 3c).

#### 2.2.2. Hypoxia/Stress-Associated Mediators Linked to Tumor Adaptation and Immune Modulation

**Figure 4:**
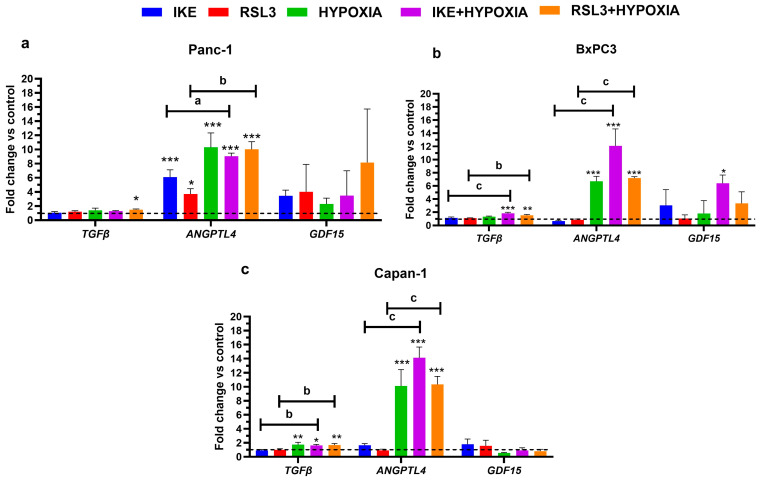
Ferroptosis induction differentially modulates immunosuppressive gene expression in PDAC cell lines (**a**) Panc-1, (**b**) BxPC3, and (**c**) Capan-1 cells under normoxic and hypoxic conditions. Relative mRNA expression of the indicated genes was evaluated following treatment with RSL3 (100 Nm) or IKE (1 µM) in normoxia or 0.1% hypoxia, measured at 36 h. Statistical significance: * *p* < 0.05, ** *p* < 0.01, *** *p* < 0.001 when compared to untreated control. ^a^ *p* < 0.05, ^b^ *p* < 0.01, ^c^ *p* < 0.001 when compared to IKE/RSL3 treated in normoxia. No symbol indicates no statistical significance.

To determine whether ferroptosis influences immunosuppressive signaling in Panc-1, BxPC3, and Capan-1 cells, we measured the changes in the gene expression for *TGFβ*, *ANGPTL4*, and *GDF1.* Distinct cell line-specific differences were observed in the regulation of immunosuppressive genes following ferroptosis induction. In Panc-1 cells, *TGFβ* expression remained largely unchanged across normoxic and hypoxic conditions following treatment with RSL3 or IKE. In contrast, both *GDF15* and *ANGPTL4* were significantly upregulated following ferroptosis induction. Increased expression was observed in response to RSL3, IKE, and hypoxia alone, with the highest induction detected in the combined hypoxia + RSL3 or hypoxia + IKE treatment groups. These findings indicate that hypoxic stress synergizes with ferroptosis to enhance the expression of stress-associated immunomodulatory genes in Panc-1 cells (Figure 4a).

BxPC3 cells demonstrated a partially similar response pattern. *TGFβ* expression remained relatively basal across all treatment groups, while *ANGPTL4* expression was markedly elevated specifically in the hypoxia + ferroptosis groups. *GDF15* expression was also increased following ferroptosis induction; however, the magnitude of induction was lower than that observed in Panc-1 cells (Figure 4b).

In Capan-1 cells, *TGFβ* and *GDF15* expression remained comparable to that of untreated control cells across all treatment groups, indicating minimal transcriptional responsiveness to ferroptotic or hypoxic stress. Similarly, no substantial induction of *ANGPTL4* was detected, suggesting that Capan-1 cells maintain a relatively stable immunosuppressive profile despite the induction of ferroptosis (Figure 4c).

#### 2.2.3. Inflammatory and Stromal-Associated Transcriptional Response Cytokines with Dual-Role Mediators That Are Context-Dependent

**Figure 5:**
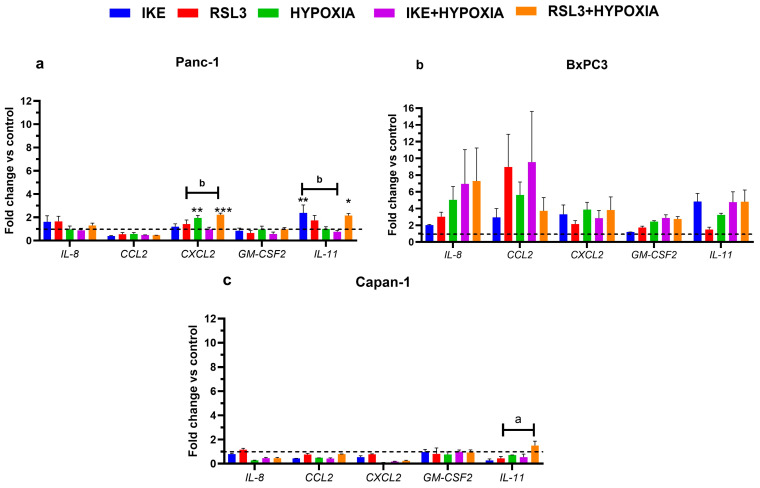
Ferroptosis differentially regulates inflammatory cytokine and chemokine expression in PDAC cells (**a**) Panc-1, (**b**) BxPC3, and (**c**) Capan-1 under normoxia and hypoxia. Relative mRNA expression of the indicated genes was evaluated following treatment with RSL3 (100 nM) or IKE (1 µM) in normoxia or 0.1% hypoxia, measured at 36 h. Statistical significance: * *p* < 0.05, ** *p* < 0.01, *** *p* < 0.001 when compared to untreated control. ^a^ *p* < 0.05, ^b^ *p* < 0.01, when compared to IKE/RSL3 treated in normoxia. No symbol indicates no statistical significance.

To further characterize the inflammatory and stromal-associated transcriptional response induced by ferroptosis, Panc-1, BxPC3, and Capan-1 cells were treated with RSL3 or IKE under normoxic and hypoxic conditions, followed by analysis of the gene expression of *IL-8*, *CCL2*, *CXCL2*, *GM-CSF (CSF2)*, and *IL-11*. Distinct cell line-specific responses were observed following ferroptosis induction. In Panc-1 cells, ferroptosis induction resulted in selective modulation of inflammatory mediators. Expression of *GM*-*CSF* (*CSF2*) and *CCL2* was consistently downregulated following treatment with RSL3 or IKE. Under normoxic conditions, *IL-8* expression increased modestly following ferroptosis induction; however, it decreased under hypoxic conditions. In contrast, combined hypoxia + RSL3 treatment induced significant upregulation of *CXCL2* and *IL-11*, suggesting activation of specific inflammatory and stress-associated pathways under hypoxic ferroptotic stress (Figure 5a).

BxPC3 cells exhibited the strongest and broadest inflammatory response among the three cell lines. Expression of *IL-8*, *CCL2*, *CXCL2*, *GM-CSF*, and *IL-11* was upregulated following ferroptosis induction under both normoxic and hypoxic conditions, indicating robust activation of inflammatory cytokine and chemokine signaling pathways in response to ferroptotic stress (Figure 5b).

Capan-1 cells showed minimal transcriptional alterations across most treatment groups, with *IL-8*, *CCL2*, *CXCL2*, *and GM-CSF* expression levels remaining comparable to untreated controls. However, *IL-11* expression was selectively upregulated in the hypoxia + RSL3 treatment group, indicating limited activation of stress-associated inflammatory signaling under combined ferroptotic and hypoxic stress (Figure 5c).

## 3. Discussion

The present study demonstrates that PDAC cell lines exhibit marked heterogeneity in their susceptibility to ferroptosis under normoxic and hypoxic conditions, accompanied by distinct innate immune and inflammatory transcriptional responses. Among the three PDAC models examined, Panc-1 cells were highly sensitive to ferroptosis induction by both RSL3 and IKE, whereas Capan-1 cells displayed profound resistance, particularly under hypoxia. BxPC3 cells exhibited an intermediate phenotype. Importantly, ferroptosis induction was associated with substantial remodeling of interferon signaling, inflammatory cytokine expression, and hypoxia-associated stress pathways, suggesting that ferroptosis in PDAC extends beyond lipid peroxidation–mediated cell death and actively shapes tumor–immune communication within the hypoxic TME.

Ferroptosis is a regulated, iron-dependent form of cell death driven by the accumulation of phospholipid hydroperoxides following the failure of antioxidant systems such as GPX4 and the system Xc^−^ [7,8]. RSL3 directly inhibits GPX4, whereas IKE inhibits the cystine/glutamate antiporter system Xc^−^ through SLC7A11 blockade. The greater cytotoxicity observed with RSL3 compared to IKE in the present study is mechanistically consistent with direct GPX4 inhibition, bypassing compensatory cystine uptake or metabolic adaptation pathways. The profound resistance of Capan-1 cells suggests alternative antioxidant defense mechanisms, potentially involving FSP1-CoQ10 signaling, DHODH-mediated mitochondrial lipid repair, NRF2 activation, or altered fatty acid metabolism [30,31]. Previous studies have shown that ferroptotic sensitivity in PDAC is strongly influenced by KRAS status, lipid composition, and oxidative stress buffering capacity, all of which are likely to contribute to the differential responses observed among Panc-1, BxPC3, and Capan-1 cells [32].

Hypoxia represents a defining feature of PDAC biology and profoundly alters metabolic and immune signaling pathways. The current findings demonstrate that hypoxia modulates ferroptosis susceptibility in a cell line-dependent manner. Panc-1 cells retained strong ferroptosis sensitivity under hypoxia, whereas BxPC3 cells became partially resistant, and Capan-1 cells became highly refractory to ferroptotic death. Hypoxia has previously been shown to exert dual effects on ferroptosis. On the one hand, HIF-1α–mediated metabolic reprogramming can suppress ROS accumulation by increasing glycolysis, reducing mitochondrial respiration, enhancing glutathione synthesis, and remodeling lipids, thereby promoting ferroptosis resistance [33]. On the other hand, hypoxia may exacerbate lipid peroxidation and iron dysregulation under severe oxidative stress, thereby potentiating ferroptosis in susceptible cells. The sustained ferroptotic sensitivity of Panc-1 cells under hypoxia may therefore reflect an inability to compensate for oxidative lipid damage despite sufficient hypoxic adaptation.

Ferroptosis is increasingly recognized as an immunologically relevant form of regulated cell death that can modulate antitumor immunity by releasing damage-associated molecular patterns (DAMPs), inflammatory mediators, and tumor-derived cytokines. Ferroptotic tumor cells can activate innate immune responses by promoting the secretion of immune-stimulatory factors, including type I IFNs, which regulate immune cell recruitment and activation [34]. Activation of IFN signaling may occur through the accumulation of endogenous nucleic acid species resulting from ferroptosis-induced oxidative stress and DNA damage, thereby activating innate immune sensing pathways [35]. In addition, ferroptosis-associated lipid peroxidation products can function as signaling molecules that influence inflammatory responses within the TME [36,37]. In PDAC, where hypoxia is a prominent feature of the TME, oxygen deprivation can reshape ferroptosis sensitivity by altering redox metabolism, antioxidant capacity, and interferon-related stress responses. Therefore, the observed modulation of IFN signaling following IKE- or RSL3-induced ferroptosis under normoxic and hypoxic conditions in Capan-1, BxPC-3, and PANC-1 cells suggests that ferroptotic stress can regulate tumor-intrinsic immune signaling pathways.

One of the most significant observations in this study is the robust activation of innate immune and interferon-associated signaling pathways following ferroptosis induction, particularly in Panc-1 cells. Ferroptosis induced substantial upregulation of *RIG-I, MAVS, IRF1, IRF3, IRF7, IRF9, STAT1, STAT2, OAS1, MX1, CXCL10,* and *ADAR-p150*, especially under hypoxia. These findings support emerging evidence that ferroptotic stress can generate immunogenic signals that activate antiviral and innate immune pathways [38]. Lipid peroxidation products, oxidized nucleic acids, mitochondrial stress, and DAMP release may activate cytosolic RNA-sensing pathways via RIG-I/MAVS signaling, ultimately triggering interferon transcriptional programs [39].

The transcriptional upregulation of *CXCL10* in Panc-1 cells is particularly noteworthy. CXCL10 is a potent chemoattractant for activating CD8+ T cells, NK cells, and Th1 lymphocytes through CXCR3 signaling [40]. Increased *CXCL10* expression may therefore enhance recruitment of cytotoxic immune cells into TME, potentially increasing anti-tumor immunity. Similarly, activation of *STAT1*, *IRF1*, and *IRF7* is associated with enhanced antigen presentation, antiviral defense, and pro-inflammatory immune activation. These findings suggest that ferroptosis in Panc-1 cells may create a partially immune-stimulatory microenvironment despite the otherwise immunosuppressive nature of PDAC.

However, the concurrent induction of stress-associated immunomodulatory genes, such as *ANGPTL4* and *GDF15*, suggests that ferroptosis may simultaneously activate compensatory immunosuppressive mechanisms. ANGPTL4 is a hypoxia-responsive factor regulated by HIF-1α and PPAR signaling that promotes vascular permeability, metastasis, extracellular matrix remodeling, and immune suppression [27]. Increased *ANGPTL4* expression under hypoxia combined with ferroptosis may contribute to tumor adaptation and macrophage polarization toward an immunosuppressive phenotype [41]. GDF15 has similarly been implicated in immune evasion, cachexia, T-cell dysfunction, and suppression of dendritic-cell activity [42]. Thus, although ferroptosis activates innate immune pathways, hypoxia-associated stress responses may simultaneously restrain effective anti-tumor immunity.

The differential inflammatory cytokine responses observed across PDAC cell lines further highlight the complex relationship between ferroptosis and immune TME. Panc-1 cells exhibited downregulation of *CCL2* and *GM-CSF* following ferroptosis induction. *CCL2* is a major recruiter of monocytes and tumor-associated macrophages (TAMs), particularly immunosuppressive M2-like macrophages. Reduced *CCL2* expression may therefore decrease recruitment of suppressive myeloid populations and partially favor anti-tumor immunity [43]. Similarly, GM-CSF has dual roles in cancer but is often associated with the expansion of myeloid-derived suppressor cells (MDSCs) in PDAC. Downregulation of GM-CSF may therefore reduce immunosuppressive stromal remodeling [44].

In contrast, BxPC3 cells demonstrated broad upregulation of *IL-8, CCL2, CXCL2, GM-CSF*, and *IL-11* following ferroptosis induction. This inflammatory phenotype suggests activation of NF-κB–associated stress signaling pathways. IL-8 and CXCL2 are potent neutrophil chemoattractants and may promote recruitment of tumor-associated neutrophils, which frequently exhibit pro-tumorigenic and immunosuppressive functions in PDAC [45,46]. Elevated IL-8 signaling has also been associated with angiogenesis, epithelial–mesenchymal transition, metastasis, and resistance to therapy [46]. Likewise, IL-11 activates STAT3 signaling and contributes to stromal fibrosis, tumor progression, and immune suppression [47]. Therefore, although ferroptosis may stimulate inflammatory signaling, the specific cytokine milieu it generates may ultimately determine whether immune activation or suppression predominates within the TME.

The minimal transcriptional responsiveness observed in Capan-1 cells likely reflects intrinsic resistance to ferroptosis and limited activation of oxidative stress signaling pathways. The selective induction of *IRF9* and *IL-11* under hypoxia + RSL3 conditions suggests that even resistant cells may activate restricted stress-response programs without fully engaging inflammatory or interferon signaling networks [47]. Such resistance may contribute to immune evasion and persistence within hypoxic tumor regions.

Taken together, our findings suggest that PDAC cell lines have varied sensitivities to the ferroptosis inducers, IKE and RSL3. The qPCR data revealed that the PDAC cell lines differ in their gene expression patterns associated with type I IFN signaling, hypoxia-associated stress, and context-dependent inflammatory mediators. These results reinforce the idea that ferroptosis is not only a process of destroying tumor cells but also a modulator of immune-related signaling pathways, potentially affecting how tumors interact with the immune system. Although hypoxia induced distinct transcriptional responses among PDAC cell lines treated with IKE/RSL3, future studies employing transcriptomic, proteomic, and functional approaches will be required to delineate the downstream regulatory networks underlying these cell line-specific responses. Recent studies suggest that ferroptosis induction may synergize with immunotherapy by increasing tumor immunogenicity and promoting recruitment of cytotoxic lymphocytes. CD8+ T cells can themselves induce ferroptosis through IFNγ-mediated suppression of SLC7A11 and SLC3A2, thereby enhancing anti-tumor activity [48]. However, the present data indicate that hypoxia and cell-specific inflammatory programs may substantially alter these outcomes. While Panc-1-like tumors may respond favorably through induction of interferon and CXCL10 signaling, BxPC3-like tumors may generate inflammatory mediators that instead promote neutrophilic inflammation, stromal remodeling, and immunosuppression.

To our knowledge, the regulation of tumor-intrinsic type I IFN signaling following pharmacological induction of ferroptosis with IKE or RSL3 in PDAC cells under hypoxia remains underexplored. While previous studies have primarily focused on how hypoxia alters ferroptosis susceptibility and execution in cancer cells [49,50]. The present study addresses an understudied aspect of ferroptosis biology by examining the tumor-intrinsic signaling consequences of ferroptotic stress. Our data suggest that ferroptosis functions not only as a mechanism of cell death but also as a regulator of innate immune-related transcriptional programs within tumor cells. Such ferroptosis-induced immune signaling responses may represent an additional layer of regulation that could influence tumor–immune interactions and the therapeutic response to immunotherapy. We acknowledge that the limitation of our current study is that the results are based on in vitro PDAC cell lines. This study does not directly assess immune cell activation. Future research using immune-competent models and functional immune profiling is needed to determine whether these tumor-intrinsic interferon responses lead to different tumor–immune interactions. Therefore, effective therapeutic exploitation of ferroptosis in PDAC will likely require combinatorial approaches targeting both oxidative stress and the hypoxic immune microenvironment.

## 4. Materials and Methods

### 4.1. Chemicals and Reagents

Imidazole Ketone Erastin, IKE (Cat. No. HY-114481), was procured from MedChem Express, Monmouth Junction, NJ, USA; Liproxstatin-1 (Cat. No. 6113) and RSL3 (Cat. No. 6118) were procured from Tocris Bioscience, Minneapolis, MN, USA. Neutral red dye (Cat. No. N7005) was procured from Sigma-Aldrich, St. Louis, MO, USA. All other chemicals, unless otherwise mentioned, were procured from Thermo Fisher Scientific, Waltham, MA, USA. Primers for qPCR were procured from Integrated DNA Technologies, Coralville, IA, USA. The qPCR primer sequences are listed in Supplementary Table S1.

### 4.2. Cell Lines and Maintenance

Human-derived PDAC cell lines— BxPC3, PANC-1, and Capan-1—were used in the present study. These cell lines were procured from ATCC and maintained in the laboratory in accordance with their guidelines. Cells were cultured in DMEM supplemented with glutamine, 10% FBS, and 1 × penicillin/streptomycin, plus 0.25 µg/L amphotericin B, in a 5% humidified CO_2_ incubator (ESCO Cell Culture incubator, Horsham, PA, USA) maintained at 37 °C. All the cell lines were routinely checked for mycoplasma contamination. Exponentially growing cells were used for the study.

### 4.3. Drug Treatment in Normoxia and Hypoxia

In this study, ferroptosis was induced using the well-known chemical agents RSL3 and imidazole ketone erastin (IKE). Both compounds were dissolved in DMSO at 50 mM and further diluted with growth media to obtain various working concentrations. Liproxstatin-1 (LIP) was dissolved in DMSO at a concentration of 10 mM and further diluted in growth media. To induce hypoxia, cells were incubated in a humidified Whitley H35 Hypoxystation (Don Whitley Scientific Limited, Bingley, West Yorkshire, UK) at 37 °C with an atmospheric composition of 94.9% N_2_, 5% CO_2_, and 0.1% O_2_. To validate the establishment of the hypoxic model, we measured HIF-1α protein expression separately (Supplementary Figure S1). HIF-1α stabilization is a well-established and widely used molecular indicator of cellular hypoxic response, as oxygen deprivation inhibits HIF-1α hydroxylation and degradation, resulting in its accumulation. Increased HIF-1α protein levels following exposure to 0.1% oxygen conditions confirmed successful induction of hypoxia in our experimental system.

### 4.4. Changes in Viability upon Treatment with RSL3/IKE in Normoxia and Hypoxia

Briefly, 3 × 10^4^ cells were plated onto a 96-well plate and allowed to attach overnight. Cells were next treated with different concentrations (90 nM–25 µM) of IKE/RSL3 or cotreated with LIP (500 nM) and incubated in normoxia or 0.1% hypoxia for 72 h, followed by assessment of cell viability by neutral red assay as described earlier [51]. LIP specifically prevents ferroptosis by inhibiting lipid peroxidation. Unlike broad-spectrum antioxidants, it exclusively targets ferroptotic pathways without affecting other cell death types such as apoptosis or necroptosis, thereby establishing itself as an effective rescue agent for ferroptosis [52]. In our experiments, we used LIP to confirm that IKE/RSL3 treatment induces ferroptotic cell death.

### 4.5. Changes in the Gene Expression for Innate Immune-Related Markers

Cells were seeded at 5 × 10^5^ cells per 60-mm dish in 4 mL of growth medium and allowed to attach overnight. Next, cells were treated with 100 nM RSL3 or 1 µM IKE for 36 h under normoxia or 0.1% hypoxia. Total RNA was then extracted using the easy-BLUE Total RNA Extraction Kit (iNtRON Biotechnologies, Gyeonggi-do, Republic of Korea) according to the manufacturer’s protocol. RNA samples were quantified using a Nanodrop. cDNA was synthesized from 1 μg of total RNA using the High-Capacity cDNA Reverse Transcription Kit (Applied Biosystems, Thermo Fisher, Waltham, MA, USA). qPCR was performed using the Maxima SYBR Green/ROX qPCR Master Mix (Thermo Scientific, Waltham, MA, USA) on a Bio-Rad Real-Time thermocycler (CFX384 Touch Real-Time PCR Detection System, Bio-Rad, CA, USA), and relative mRNA expression was normalized to β-actin.

### 4.6. Statistical Analysis

Statistical analyses for all assays were performed using GraphPad Prism, version 5.0 (GraphPad Software, San Diego, CA, USA). For all comparisons, either an unpaired (two-tailed) *t*-test or a one-way analysis of variance (ANOVA) with Bonferroni’s post hoc test was used. A *p*-value of <0.05 was considered statistically significant. Results are presented as mean ± SD from three independent experiments.

## 5. Conclusions

In conclusion, the present study demonstrates that inducing ferroptosis in PDAC elicits highly heterogeneous responses that are profoundly shaped by hypoxia and intrinsic cellular context. Panc-1 cells exhibited strong sensitivity to ferroptosis, accompanied by robust activation of interferon-associated innate immune pathways, whereas BxPC3 cells displayed a predominantly inflammatory cytokine response, and Capan-1 cells remained largely resistant to both ferroptosis and transcriptional reprogramming. These findings indicate that ferroptosis is not solely a cell death mechanism but also an important regulator of tumor–immune communication within the PDAC microenvironment. While hypoxia amplified IFN signaling and *CXCL10* expression in sensitive cells, it also promoted expression of stress-associated immunomodulators, such as *ANGPTL4* and *GDF15*, which may contribute to immune suppression and tumor adaptation. The balance between these opposing inflammatory and immunosuppressive programs likely determines whether ferroptosis promotes anti-tumor immunity or facilitates tumor progression. Future research employing single-cell and spatial transcriptomics will be necessary to determine whether hypoxia-related ferroptotic responses and interferon signaling pathways are localized within tumor subpopulations or are influenced by interactions with stromal and immune cells.

## Data Availability

The data presented in this study are included in the article. Further inquiries can be directed to the corresponding author.

## Supplementary Materials

The following supporting information can be downloaded at: https://www.mdpi.com/article/10.3390/ijms27146397/s1.

## Abbreviations

The following abbreviations are used in this manuscript:

ADAR-p150	Adenosine Deaminase Acting on RNA 1, p150 Isoform
ANGPTL4	Angiopoietin-like 4
CCL2	C-C Motif Chemokine Ligand 2
CXCL10	C-X-C Motif Chemokine Ligand 10
CXCL2	C-X-C Motif Chemokine Ligand 2
GDF15	Growth Differentiation Factor 15
GM-CSF	Granulocyte-Macrophage Colony-Stimulating Factor
HIF-1α	Hypoxia-inducible factor 1 alpha
IFN	Interferon
IKE	Imidazole ketone erastin
IL-11	Interleukin-11
IL-8	Interleukin- 8
IRF1	Interferon Regulatory Factor 1
IRF3	Interferon Regulatory Factor 3
IRF7	Interferon Regulatory Factor 7
IRF9	Interferon Regulatory Factor 9
LIP	Liproxstatin-1
MAVS	Mitochondrial Antiviral Signaling Protein
MX1	MX Dynamin Like GTPase 1
OAS1	2′-5′-Oligoadenylate Synthetase 1
PDAC	Pancreatic Ductal Adenocarcinoma
RIG-I	Retinoic Acid-Inducible Gene I
RSL3	RAS-selective lethal 3
STAT1	Signal Transducer and Activator of Transcription 1
STAT2	Signal Transducer and Activator of Transcription 2
TGFβ	Transforming Growth Factor Beta 1
TME	Tumor microenvironment
