# Supplementary material for: Hypoxia Differentially Regulates Ferroptosis Sensitivity and Tumor Cell-Intrinsic Type I Interferon Signaling in Pancreatic Ductal Adenocarcinoma Cells
Source: Int J Mol Sci. 2026 Jul 18;27(14):6397. doi: 10.3390/ijms27146397 (PMC13410365; doi:10.3390/ijms27146397)
Supplement: Supplementary file 1 [file ijms-27-06397-s001.zip › ijms-4384048-supplementary.pdf]

| Gene name | SEQUENCES |
|---|---|
| β Actin -F | TCCTTCCTGGGCATGGAGT |
| β Actin -R | AGCACTGTGTTGGCGTACAG |
| IRF1-F | GCTGGGACATCAACAAGGAT |
| IRF1-R | TGGTCTTTCACCTCCTCGAT |
| IRF3-F | AAGAAGGGTTGCGTTTAGCA |
| IRF3-R | TCCAGAATGTCTTCCTGGGTA |
| IRF7-F | CATCTTCAAGGCCTGGGCTG |
| IRF7-R | TTATCCCGCAGCATCACGAA |
| IRF9-F | CTGCGCTTGTGTGTTGCATA |
| IRF9-R | GGCCCTGAAAGTACCTGACC |
| OAS1-F | TCCGTGAAGTTTGAGGTCCAG |
| OAS1-F | AGGTTTATAGCCGCCAGTCAA |
| MX1-F | GGAGGCACTGTCAGGAGTTG |
| MX1-R | TCCTGGTAACTGACCTTGCC |
| MAVS-F | CCAGCACCATCCAAATTGCC |
| MAVS-R | CTCATTTCTGCTGCTCCCGT |
| RIG1-F | GAAGATCCAGAATGCCAGAATC |
| RIG1-R | CCACAACCTGTAGGAGCACA |
| STAT1-F | TACACCTACGAACATGACCCT |
| STAT1-R | TCACCAACAGTCTCAACTTCAC |
| STAT2-F | CCCGCTGACTGAAATCATCC |
| STAT2-R | AGTTCATCCACCTGTCTATTAGAG |
| ADAR-p150-F | CTTCCAGTGCGGAGTAGCG |
| ADAR-p150-R | GTGACGGTGTCTGCTTTCCA |
| CXCL10-F | GTGGCATTCAAGGAGTACCTC |
| CXCL10-R | TGATGGCCTTCGATTCTGGATT |
| TGFϐ-F | ATGACCCTCACCTCTATGTACC |
| TGFϐ-R | CACAGTTCACAGTTACAATCCCA |
| GDF15-F | CTGCTTACAGGGTCTGGTGT |
| GDF15-R | CACCTGGGGTCTGGCTTTAT |
| ANGPTL4-F | CCTCTCCGTACCCTTCTCCA |
| ANGPTL4-R | AAACCACCAGCCTCCAGAGA |
| IL8-F | TGTAAACATGACTTCCAAGC |
| IL8-R | AAAACTGCACCTTCACAC |
| CCL2-F | CCCAAGCAGAAGTGGGTTCA |
| CCL2-R | GTGTCTGGGGAAAGCTAGGG |
| CXCL2-F | TCACAGTGTGTGGTCAACATTTC |
| CXCL2-R | TCTCTGCTCTAACACAGAGGGA |
| IL11-F | GCGGACAGGGAAGGGTTAAA |
| IL11-R | GCGGCAAACACAGTTCATGT |
| GM-CSF2-F | ACTTCCTGTGCAACCCAGATT |
| GM-CSF2-R | CAGCAGTCAAAGGGGATGACA |

**Supplementary Table S1-** List of primers with sequences used for qPCR

**Supplementary Figure S1**

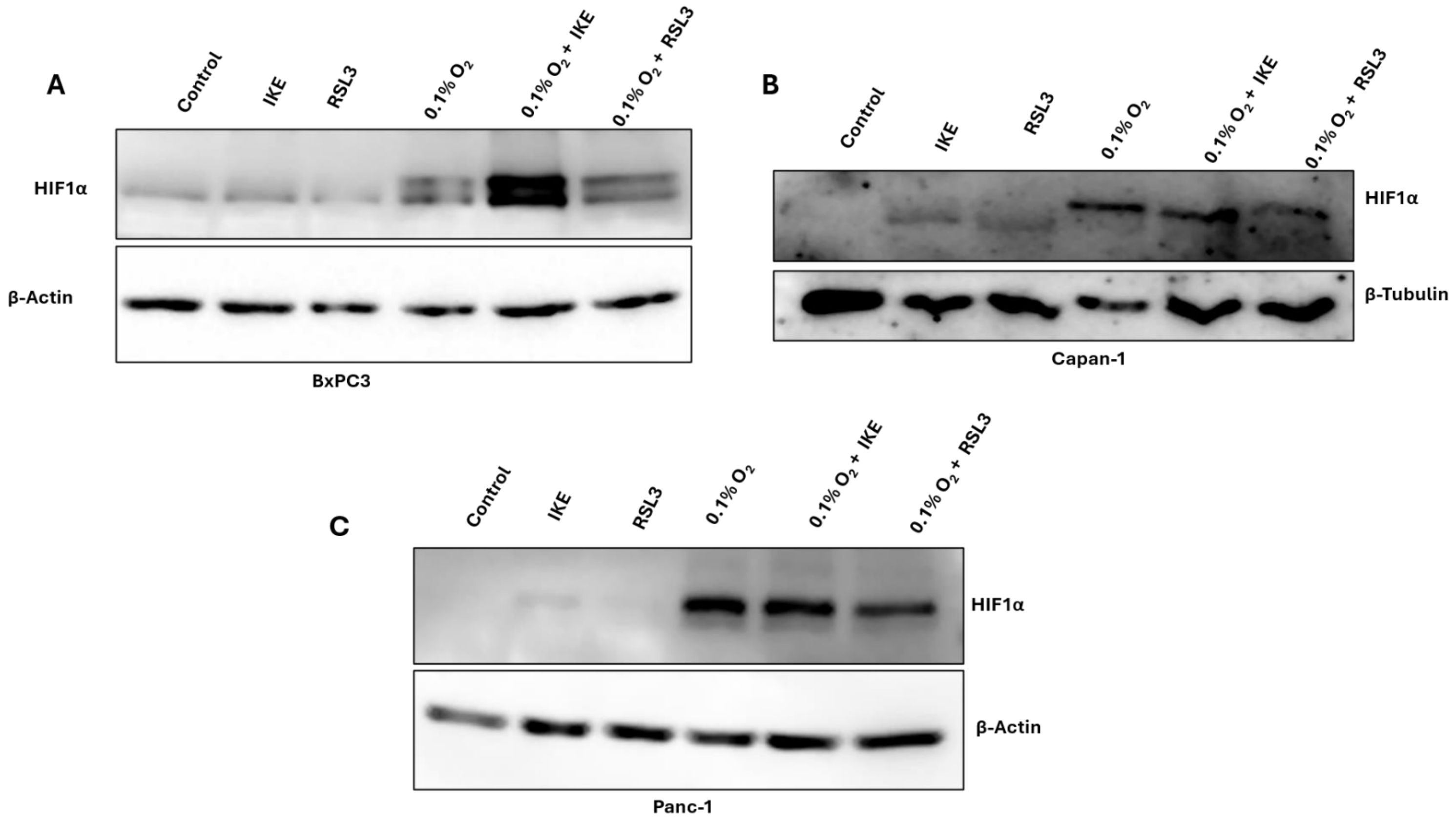


**Supplementary Figure S1-** Representative western blot analysis of changes in the expression of HIF1α in different PDAC cells (**A-** BxPC3**; B-** Capan-1 and **C-** Panc-1**)** when treated with IKE/RSL3 in normoxia or hypoxia -0.1% $O_2$ as measured at 36 h. β-Actin/βtubulin served as loading controls

Briefly, all three cell lines (BxPC3, Capan-1, and Panc-1) were treated with IKE/RSL3 (1 µM/100 nM) for 36 h under normoxic or hypoxic conditions, and cells were then collected for protein isolation. Cells were lysed in standard RIPA buffer containing 1.0% Triton X-100, 0.1% SDS, 1 mM sodium orthovanadate, and 1x protease/phosphatase inhibitor. Lysates were prepared by centrifugation at 14000 rpm for 10 min at 4 °C. Lysates were quantified using the standard BCA method, and 50 µg of protein was loaded onto 8% SDS-PAGE gels. The resolved proteins were electroblotted onto nitrocellulose membranes. The nitrocellulose membrane was further blocked with 5% skimmed milk in 0.1% TBST (Tris-buffered saline, pH 7.4, containing Tween-20). Next, the membranes were probed with primary antibodies for HIF1a (#14179; Cell Signaling Technology; 1:1500) or β-Actin (sc-47778; Santa Cruz Biotechnology) / β-tubulin (#2146, Cell Signaling Technology; 1:8000), prepared in 0.1% TBST, overnight at 4 °C with gentle rocking.

Next, membranes were washed and incubated with secondary antibody - Goat anti-rabbit (#7074; Cell Signaling Technology) or anti-mouse (#7076; Cell Signaling Technology) IgG (H+L)-HRP conjugated at a dilution of 1:12000 for 1 h at room temperature. Membranes were then developed using the SuperSignal West Pico PLUS Chemiluminescent Substrate kit (Thermo Fisher Scientific, USA), and chemiluminescence was detected with the iBright CL750 Imaging System (Thermo Fisher Scientific, USA).

Data presented in Supplementary Figure S1 demonstrate that cells exposed to 0.1% oxygen conditions exhibited accumulation of HIF-1α, a well-established marker of cellular hypoxic response. This increase in HIF-1α protein levels confirms the activation of hypoxia signaling and validates that the experimental conditions successfully induced a hypoxic state in the cells.
